# Collaborative strategies for deploying AI-based physician decision support systems: challenges and deployment approaches

**DOI:** 10.1038/s41746-023-00889-6

**Published:** 2023-08-05

**Authors:** Mirja Mittermaier, Marium Raza, Joseph C. Kvedar

**Affiliations:** 1 Charité - Universitätsmedizin Berlin, Corporate Member of Freie Universität Berlin and Humboldt-Universität zu Berlin, Department of Infectious Diseases, Respiratory Medicine and Critical Care, Berlin, Germany; 2 Berlin Institute of Health at Charité—Universitätsmedizin Berlin, Charitéplatz 1, 10117 Berlin, Germany; 3 Harvard Medical School, Boston, MA USA

**Keywords:** Diagnosis, Prognosis

## Abstract

AI-based prediction models demonstrate equal or surpassing performance compared to experienced physicians in various research settings. However, only a few have made it into clinical practice. Further, there is no standardized protocol for integrating AI-based physician support systems into the daily clinical routine to improve healthcare delivery. Generally, AI/physician collaboration strategies have not been extensively investigated. A recent study compared four potential strategies for AI model deployment and physician collaboration to investigate the performance of an AI model trained to identify signs of acute respiratory distress syndrome (ARDS) on chest X-ray images. Here we discuss strategies and challenges with AI/physician collaboration when AI-based decision support systems are implemented in the clinical routine.

## Potential roles for AI-based algorithms in clinical settings

In a recent New York Times editorial, an author dubbed artificial intelligence (AI) a “Pandora’s box” that humans were lifting the lid of^1^. Indeed, AI seems to be everywhere, from the realistic appearing conversations of ChatGPT to the image recognition software allowing many of us to unlock our cellphones with a glance. Within healthcare, both excitement and concern about AI are nothing new.

Over the last years, several AI-based models have demonstrated their usefulness in various medical disciplines, including dermatology^2^, ophthalmology^3^, and radiology^4^. Thus far, the prevailing thought within healthcare has been that AI will be most useful within clinical decision support systems (CDSS). CDSS aim to aid clinicians with the complex decision-making process to improve healthcare quality. Where exactly AI fits best within the decision-making process remains debated^5^.

In a recent case study, Farzaneh et al.^5^ systematically determined how AI could be integrated into a specific clinical scenario. They took an AI model^6^ trained to identify patterns of acute respiratory distress syndrome (ARDS) on chest X-ray images and evaluated its strengths and weakness compared to physicians. In doing so, they tested four different physician–AI collaboration strategies. One strategy involved the AI model reviewing a chest X-ray first and then deferring to a physician in cases of uncertainty. This strategy achieved higher diagnostic accuracy compared to the other three, which included physicians reviewing the X-ray first and deferring to the AI model in cases of uncertainty, the AI model examining the X-ray alone, or the physician examining the X-rays alone.

Ultimately, these findings imply that the AI model had higher and more consistent accuracy on less complicated chest X-rays, while physicians had higher accuracy on difficult chest X-rays^5^. This could mean that those caring for ARDS patients can use AI models to help triage these patients when the X-ray findings are clear, while physicians focus on interpreting the more complicated images.

## Key challenges hampering CDSS implementation into clinical practice

Despite the promises of AI-based CDSS, obstacles are blocking its implementation. Four key challenges to the widespread use of AI-based CDSS include trust, bias, scalability, and deployment.

### Trust

Both patients and clinicians must trust AI models used for decision support if they are to be widely adopted^7,8^. This study by Farzaneh et al. ^5^ is an example of the type of research that must continue to be done to evaluate and build trust in AI/physician collaboration workflows. Additionally, AI models will need to be explainable, describing why and which parameters impacted a model’s decision. For example, visualizing alerts or displaying regions of concern in an X-ray can help to overcome the distrust in the “black-box” nature of most AI-based systems^8,9^. On the other hand, excessive trust and reliance on a CDSS can interfere with developing clinical skills^10^.

### Bias

Datasets used to train AI models can contain bias, amplifying existing inequity in the healthcare system. This type of bias specifically harms disadvantaged populations^11,12^. For example, AI models trained on a dataset with unequal representation of a particular minority group might generate less accurate predictions for that minority patient population, leading to worse patient care. Various strategies for detecting and mitigating bias^12,13^ have been developed to tackle this issue, but further approaches are pivotal for generalizability and fairness.

### Scalability

While Farzaneh et al.^5^ describe a specific case study that helps optimize AI-physician collaboration, it is unrealistic to expect every implementation of AI support to occur only after a published trial. Instead, AI-CDSS will likely scale based on inferences from studies of similar clinical challenges. The specific point at which AI will be implemented in a workflow will ultimately differ among healthcare settings.

Looking into the future, generalizable CDSS tools will be implemented in healthcare settings in which they were not actually developed. The details surrounding how such generalizable tools will be developed and implemented in local workflows remain up to question^14^. Other challenges include insufficient IT infrastructure in under-resourced clinical settings where building on AI is challenging. As technical complexity increases, increased computer literacy and proficiency will be required. Lacking these skills can be hindering for clinical decision support systems adoption^10,15^.

### Deployment

Challenges to AI-based CDSS on the deployment level are centered around regulatory concerns and long-term effects. AI-based CDSS will require new rules around where responsibility lies for potential mistakes. To take just one example, not using physician decision support systems could be considered malpractice by an individual physician, or a healthcare institution that has adopted the AI-CDSS tool^16^.

## AI in medical education

AI as well as augmented and virtual reality offer unique opportunities for medical training and education^17^. For example, for medical students AI tools could provide a three-dimensional virtual reality experience that can change the way of Anatomy teaching and learning^18^. Further, as medical training during the COVID-19 pandemic became challenging, surgeon training in lung cancer surgery through the metaverse (a 3-D-enabled digital space using augmented and virtual reality) was implemented in a smart operating room^19^. Other recent publications demonstrated how AI could be used to identify a surgeon’s skill^20–22^ and thus improve continuous learning. Integrating AI in medical (student) education may not only enrich the teaching and learning experience, but may also help to teach opportunities and challenges of AI, and thus results in more awareness, trust, and better use of AI-based systems.

## Conclusion

In many cases, CDSS demonstrated a better outcome if AI algorithms collaborated with physicians. However, integrating CDSS into the daily clinical routine using real-world data requires rigorous clinical validation in a real-world environment before implementation in clinical practice. Key challenges include trust, bias, scalability, and deployment. Further, regulatory and privacy issues need to be addressed.
